# New global error bound for extended linear complementarity problems

**DOI:** 10.1186/s13660-018-1847-z

**Published:** 2018-09-24

**Authors:** Hongchun Sun, Min Sun, Yiju Wang

**Affiliations:** 1 School of Mathematics and Statistics, Linyi University, Linyi, China; 2 School of Mathematics and Statistics, Zaozhuang University, Zaozhuang, China; 3 School of Management Science, Qufu Normal University, Rizhao, China

**Keywords:** 65H10, 90C33, 90C30, ELCP, Global error bound, Residual function

## Abstract

For the extended linear complementarity problem (ELCP), by virtue of a new residual function, we establish a new type of global error bound under weaker conditions. Based on this, we respectively obtain new global error bounds for the vertical linear complementarity problem and the mixed linear complementarity problem. The obtained results presented in this paper supplement some recent corresponding results in the sense that they can provide some error bounds for a more general ELCP. Their feasibility is verified by some numerical experiments.

## Introduction

Consider the extended linear complementarity problem (ELCP) of finding vector $(x, y)\in R^{n}\times R^{n}$ such that

$$ Mx-Ny\in{ \mathcal {K}}, \quad x\geq0, y\geq0, x^{\top}y=0, $$

where $M,N\in R^{m\times n}$ and ${\mathcal {K}}=\{Qz+ q | z \in R^{l}\}$ with $Q\in R^{m\times l}$, $q\in R^{m}$. The solution set of the ELCP is denoted by $X^{*}$ which is assumed to be nonempty throughout this paper.

The ELCP finds applications in various domains, such as engineering, economics, finance, and robust optimizations [1, 2]. It was first considered by Mangasarian and Pang [1] and was further considered by Gowda [3] and Xiu et al. [4]. For more details on its development, see [5] and the references therein. It is well known that the global error bound plays an important role in theoretical analysis and numerical treatment of optimization problems such as variational inequalities and nonlinear complementarity problems [5–14]. The global error bound for the classical linear complementarity problems is well studied (see, e.g.,[6, 15–19]). For a class of generalized linear complementarity problems, the global error bound was fully analyzed in [20–22]. Zhang and Xiu [4] presented an error bound for the ELCP with the column monotonicity and for the $R_{0}$-ELCP. In this paper, we give a further consideration on this issue by establishing a global error bound estimation for the ELCP under a milder condition motivated by the work in [4].

## Results and discussion

Here we are concerned with the global error bound on the distance between a given point in $R^{2n}$ and the solution set of the ELCP in terms of some residual functions. This paper is a follow-up to [4], as in this paper we establish a new global error bound for the ELCP under weaker conditions than those used in [4].

Some error bounds for the ELCP have been presented in [4], and they hold under some stringent condition, that is, the underlying matrices *M*, *N* satisfy the column monotonicity with respect to ${{\mathcal {K}}}$ or $R_{0}$-property. Furthermore, we can only get the error bound of any points in the set $\Omega=\{(x,y)\in R^{2n}|Mx-Ny\in {\mathcal {K}}\}$ by the results in [4]. Then the following two questions are posed naturally: Can the conditions imposed on the matrices *M*, *N* in [4] be relaxed or removed? How about the global error bound estimation in $R^{2n}$ for the ELCP? These constitute the main topics of this paper. In this paper, we shall deal with the two issues. In fact, based on some equivalent reformulation of the ELCP and using a new type residual function, we present a global error bound for the ELCP in $R^{2n}$ under a milder condition, and the requirement of the column monotonicity, or $R_{0}$-property, or non-degenerate solution, and so on is removed here. Furthermore, the global error bounds for the vertical linear complementarity problem (VLCP) and the mixed linear complementarity problem (MLCP) are also discussed in detail.

## Methods and notations

The aim of this study is to design a new global error bound for the ELCP. More specifically, the ELCP is firstly converted into an equivalent extended complementarity problem, which eliminates the variable *z* in the ELCP. Then, we define a residual function of the transformed problem, based on which we derive some new error bounds for the transformed problem and the original ELCP. Furthermore, we deduce some global error bounds for the two special cases of the ELCP: VLCP and MLCP. Note that the obtained results can be viewed as some supplements to the results in [4].

We adopt the following notations throughout the paper. All vectors are column vectors and the superscript *T* denotes the transpose. The $x_{+}$ denotes the orthogonal projection of vector $x\in R^{n}$ onto $R^{n}_{+}$, that is, $(x_{+})_{i}:=\max\{x_{i},0\}$, $1\leq i \leq n$; the norm $\|\cdot\|$ and $\|\cdot\|_{1}$ denote the Euclidean 2-norm and 1-norm, respectively. For $x,y\in R^{n}$, use $(x;y)$ to denote the column vector $(x^{\top},y^{\top})^{\top}$, and $\min\{x,y\}$ means the componentwise minimum of *x* and *y*. We use $I_{m}$ to denote an identity matrix of order *m*, use $D^{+}$ to denote the pseudo-inverse of matrix *D*, use $\operatorname{diag}(a_{1},a_{2},\ldots,a_{n})$ to denote the diagonal matrix with elements $a_{1},a_{2},\ldots,a_{n}$. For any $n\times n$ real matrix *A*, we denote by $A^{\top}$ the transpose of *A*, by $\|A\|$ the matrix norm of *A*, that is, $\|A\|:=\max(\lambda (A^{\top}A))^{\frac{1}{2}}$, where $\lambda(A^{\top}A)$ is an eigenvalue of the matrix $A^{\top}A$, denote a nonnegative vector $x\in R^{n}$ by $x\ge0$, denote an absolute value of the real number *a* by $|a|$, and use $C^{k}_{n}$ to denote the combinatorial number, which is the number of combinations when *k* elements are arbitrarily taken from *n* elements. We denote the empty set by ∅.

## List of abbreviations

In this section, we give the following tabular for abbreviations used in this paper (see Table 1).

**Table 1:** Abbreviations in this paper

Abbreviations	Description
ELCP	The extended linear complementarity problem
VLCP	The vertical linear complementarity problem
MLCP	The mixed linear complementarity problem

## Global error bound for ELCP

In this section, we first present an equivalent reformulation of the ELCP in which parameter *z* is not involved and then establish a global error bound for the ELCP under weaker conditions.

From the definition of the ELCP, the following result is straightforward.

### Proposition 5.1

*Vector* $(x^{*};y^{*})$ *is a solution of the ELCP if and only if there exists* $z^{*}\in R^{l}$ *such that*

$$ x^{*}\geq0, y^{*}\geq0,\bigl(x^{*} \bigr)^{\top}y^{*}=0,\qquad Mx ^{*}-Ny^{*}-Qz^{*}-q=0. $$

Let $w=(x;y)$ and $U=QQ^{+}-I_{m}$. Using the fact that $x=A^{+}b$ is a solution to the linear equation $Ax=b$ if it is consistent, we conclude that the last equation in Proposition 5.1 is equivalent to

5.1 $$ U(M,-N)w-Uq=0. $$

Define block matrices $A=(I_{n}, 0_{n})$, $B=(0_{n}, I_{n})$. Then the ELCP can be equivalently reformulated as the following extended complementarity problem w.r.t. *w*:

5.2 $$ \textstyle\begin{cases} Aw\geq0,\qquad Bw\geq0, \\ (Aw)^{\top}Bw=0, \\ U(M,-N)w-Uq=0. \end{cases} $$

We denote its solution set by $W^{*}$, and let

5.3 $$ f(w)= \bigl\Vert (-w)_{+} \bigr\Vert ^{2}+\bigl[\operatorname{sgn}\bigl(w^{\top}\hat{M}w\bigr)\bigr] w^{\top}\hat{M}w+ \bigl\Vert U(M,-N)w-Uq \bigr\Vert ^{2}, $$

where

$$\hat{M}=\left(\begin{array}{cc} 0 & I\\ I & 0 \end{array}\right),\;\mathrm{sgn}\left(w^{⊤}\hat{M}w\right)=\left\{ \begin{array}{cc} 1 & \text{if }w^{⊤}\hat{M}w>0,\\ 0 & \text{if }w^{⊤}\hat{M}w=0,\\ -1 & \text{if }w^{⊤}\hat{M}w<0. \end{array}\right.$$

Then it holds that $\{w\in R^{2n} | f(w)=0\}=W^{*}$.

From the definition of $f(w)$, a direct computation yields that

$$\begin{aligned} f(w) =& \bigl\Vert (-w)_{+} \bigr\Vert ^{2}+w^{\top}\bigl[\operatorname{sgn}\bigl(w^{\top}\hat{M}w\bigr) \bigr]\hat{M} w+ \bigl\Vert U(M,-N)w-Uq \bigr\Vert ^{2} \\ =&w^{\top} \operatorname{diag}(\sigma_{1},\sigma_{2},\ldots, \sigma_{2n}) w+w ^{\top}\bigl[\operatorname{sgn}\bigl(w^{\top}\hat{M}w \bigr)\bigr]\hat{M}w \\ &{} + w^{\top}(M,-N)^{\top}U^{\top}U(M,-N)w-2q^{\top}U^{\top}U(M,-N)w+ q^{\top}U^{\top}Uq \\ =&w^{\top}\bigl\{ \operatorname{diag}(\sigma_{1},\sigma_{2}, \ldots,\sigma_{2n})+\bigl[\operatorname{sgn}\bigl(w^{\top}\hat{M}w\bigr)\bigr] \hat{M}+(M,-N)^{\top}U^{\top}U(M,-N)\bigr\} w \\ &{} -2q^{\top}U^{\top}U(M,-N)w+ q^{\top}U^{\top}Uq \\ =&w^{\top}\hat{Q} w-2q^{\top}U^{\top}U(M,-N)w+ q^{\top}U^{\top}Uq, \end{aligned}$$

where

5.4 $$ \hat{Q}:=M_{1}+M_{2}. $$

Set

5.5 $$ M_{1}=\bigl[\operatorname{sgn}\bigl(w^{\top}\hat{M}w\bigr) \bigr]\hat{M}+(M,-N)^{\top}U^{\top}U(M,-N),\qquad M_{2}= \operatorname{diag}(\sigma_{1},\sigma_{2},\ldots,\sigma_{2n}), $$

with

$$ \sigma_{i}=\textstyle\begin{cases} 1& \text{if } w_{i}>0, \\ 0& \text{if } w_{i}\leq0 \end{cases} $$

and $H= \{M_{2}\in R^{2n\times2n} | M_{2}=\operatorname{diag}(\sigma_{1}, \sigma_{2},\ldots,\sigma_{2n})\}$. Then, by the definition of $\sigma_{i}$, we can get that the cardinality of the set *H* is

$$ C^{0}_{2n}+C^{1}_{2n}+C^{2}_{2n}+ \cdots+C^{2n-1}_{2n}+C^{2n}_{2n}=2^{2n}. $$

Applying the related theory of linear algebra and (5.4), we give the following result for our analysis.

### Lemma 5.1

*For* $f(w)$ *defined in* (5.3), *there exists an affine transformation* $w=C_{1}v+p_{1}$ *with an orthogonal matrix* $C_{1} \in R^{2n\times2n}$ *and vector* $p_{1}\in R^{2n}$ *such that*

$$ f(w)=g(v):=\sum_{i\in I_{+}}a_{i}v_{i}^{2}- \sum_{i\in I_{-}}a_{i}v _{i}^{2}- \sum_{j\in J}c_{j}v_{j}+\tau, $$

*where* $a_{i}, c_{j}, \tau\in R$, $a_{i}>0$, $i\in I_{+}\cup I_{-}$, $j \in J$,

$$\begin{aligned}& I_{+}=\bigl\{ i\in\{1,2,\ldots,2n\} | \lambda_{i}>0 \bigr\} , \\& I_{-}=\bigl\{ i\in\{1,2,\ldots,2n\} | \lambda_{i}< 0\bigr\} , \\& J=\bigl\{ j\in\{1,2,\ldots,2n\} | \lambda_{j}=0\bigr\} , \end{aligned}$$

*and* $\lambda_{1},\lambda_{2},\ldots,\lambda_{2n}$ *are real eigenvalues of the matrix* *Q̂*.

### Proof

Since the matrix *Q̂* is symmetric, there exists an orthogonal matrix $C_{1}\in R^{2n\times2n}$ such that

5.6 $$ C_{1}^{\top}\hat{Q} C_{1}=\operatorname{diag}( \lambda_{1},\lambda_{2},\ldots ,\lambda_{2n}). $$

Let $w=C_{1}\xi$ and

5.7 $$ 2q^{\top}U^{\top}U(M,-N)C_{1}=(c_{1},c_{2}, \ldots,c_{2n}). $$

Then

5.8 $$\begin{aligned} f(w) =&\xi^{\top}C_{1}^{\top} \hat{Q} C_{1}\xi-2q^{\top}U^{\top}U(M,-N)C _{1}\xi+ q^{\top}U^{\top}Uq \\ =&\sum^{2n}_{i=1}\lambda_{i} \xi_{i}^{2}-\sum^{2n}_{i=1}c _{i}\xi_{i}+q^{\top}U^{\top}Uq \\ =&\sum_{i\in I_{+}\cup I_{-}}\lambda_{i}\biggl( \xi_{i}-\frac{c _{i}}{2\lambda_{i}}\biggr)^{2}-\sum _{j\in J}c_{j}\xi_{j}-\sum _{i\in I_{+}\cup I_{-}}\frac{c_{i}^{2}}{4\lambda_{i}}+q^{ \top}U^{\top}Uq. \end{aligned}$$

Let

$$ v_{i}=\textstyle\begin{cases} \xi_{i}-\frac{c_{i}}{2\lambda_{i}},&\text{if } i\in I_{+}\cup I_{-}, \\ \xi_{i}, &\text{if } i\in J. \end{cases} $$

Then there exists vector $p_{2}$ such that $v=\xi-p_{2}$. This and $w=C_{1}\xi$ imply $w=C_{1}v+p_{1}$ with $p_{1}=C_{1}p_{2}$. By (5.8), letting

5.9 $$ a_{i}=\lambda_{i}, \quad i\in I_{+},\qquad a_{i}=-\lambda_{i}, \quad i\in I_{-},\qquad \tau=-\sum_{i\in I_{+}\cup I_{-}} \frac{c_{i}^{2}}{4 \lambda_{i}}+q^{\top}U^{\top}Uq $$

yields the desired result as follows. □

In the following, we present our main error bound result for the ELCP.

### Theorem 5.1

*Suppose that* $W^{*}=\{w\in R^{2n} | f(w)=0\}$ *is nonempty*. *Then there exists a constant* $\rho_{0}>0$ *such that*, *for any* $w\in R^{2n}$, *there exists* $\bar{w}\in W^{*}$ *satisfying*

$$ \Vert w-\bar{w} \Vert \leq\rho_{0}\bigl( \bigl\vert f(w) \bigr\vert + \bigl\vert f(w) \bigr\vert ^{\frac{1}{2}}\bigr). $$

### Proof

Applying Lemma 5.1, there exists an affine transformation $w=C_{1}v+p_{1}$ such that

5.10 $$ f(w)=g(v):=\sum_{i\in I_{+}}a_{i}v_{i}^{2}- \sum_{i\in I_{-}}a_{i}v _{i}^{2}- \sum_{j\in J}c_{j}v_{j}+\tau, $$

where $a_{i}>0$, $c_{j},\tau\in R$, $i\in I_{+}\cup I_{-}$, $j\in J$ are defined in (5.9), $I_{+}$, $I_{-}$, and *J* are respectively non-overlapping subsets of $\{1,2,\ldots,2n\}$, and $C_{1}$ is an orthogonal matrix. Therefore, given $w=(w_{1},w_{2},\ldots,w_{2n})^{ \top}\in R^{2n}$, one has

$$ v=C^{\top}_{1}(w-p_{1}):=(v_{1},v_{2}, \ldots,v_{2n})^{\top}. $$

Now, we break the discussion into three cases.

*Case* 1. If $J\neq\emptyset$, we take an index $j\in J$ such that $|c_{j}|=\max\{|c_{i}| | i\in J\}>0$, and let *v̄* with entries

$$ \bar{v}_{i}=\textstyle\begin{cases} v_{i},&\text{if } i\neq j, \\ v_{j}+\frac{1}{c_{j}}g(v), &\text{if } i= j. \end{cases} $$

Then $\|v-\bar{v}\|=\frac{1}{|c_{j}|}|g(v)|$ and

$$\begin{aligned} g(\bar{v}) =&\sum_{i\in I_{+}}a_{i} \bar{v}_{i}^{2}-\sum_{i\in I_{-}}a_{i} \bar{v}_{i}^{2}-\sum_{i\in J}c_{i} \bar{v}_{i}+\tau \\ =&\sum_{i\in I_{+}}a_{i}v_{i}^{2}- \sum_{i\in I_{-}}a _{i}v_{i}^{2}- \biggl(\sum_{i\in J}c_{i}v_{i}+g(v) \biggr)+\tau \\ =&\biggl[\sum_{i\in I_{+}}a_{i}v_{i}^{2}- \sum_{i\in I_{-}}a _{i}v_{i}^{2}- \sum_{i\in J}c_{i}v_{i}+\tau \biggr]-g(v) \\ =&g(v)-g(v)=0. \end{aligned}$$

From $w=C_{1}v+p_{1}$, we get $\bar{w}=C_{1}\bar{v}+p_{1}$ and $f(\bar{w})=g(\bar{v})=0$. Thus, $\bar{w}\in W^{*}$. Furthermore,

$$\begin{aligned} \Vert w-\bar{w} \Vert =& \bigl\Vert (C_{1}v+p_{1})-(C_{1} \bar{v}+p_{1}) \bigr\Vert \\ =& \bigl\Vert C_{1}(v-\bar{v}) \bigr\Vert \\ =& \Vert v-\bar{v} \Vert \\ =&\frac{1}{ \vert c_{j} \vert } \bigl\vert g(v) \bigr\vert \\ =&\frac{1}{ \vert c_{j} \vert } \bigl\vert f(w) \bigr\vert \\ \leq&\frac{1}{ \vert c_{\min } \vert } \bigl\vert f(w) \bigr\vert , \end{aligned}$$

where the second equality follows from the fact that $C_{1}$ is an orthogonal matrix. Since the cardinality of the set *H* is $2^{2n}$, then $|c_{\min }|=\min\{|c_{j}| | j=1,2,\ldots,2^{2n}\}$, so $|c_{\min }|$ is independent of selection of the matrix $M_{2}$, i.e., $|c _{\min }|$ is independent of selection of vector *w*. Thus, the last inequality holds. The desired result is proved for this case.

*Case* 2. If $J=\emptyset$ and $\tau\leq0$ where *τ* is defined by (5.9). Let $v_{2n+1}=\sqrt{-\tau}$ and $\tilde{I}_{-}=I_{-}\cup\{2n+1\}$. Then

5.11 $$ g(v)=\bar{g}(v,v_{2n+1}):=\sum _{i\in I_{+}}a_{i}v_{i}^{2}- \sum _{i\in\tilde{I}_{-}}a_{i}v_{i}^{2}, $$

where $a_{i}\in R$, $a_{i}>0$, $i\in I_{+}\cup I_{-}$, $a_{2n+1}=1$, and $I_{+}$, $\tilde{I}_{-}$ are respectively non-overlapping subsets of $\{1,2,\ldots,2n,2n+1\}$. Let

$$ z_{i}=\textstyle\begin{cases} \sqrt{a_{i}}v_{i},& i\in I_{+}\cup I_{-}, \\ v_{i}& \text{otherwise}. \end{cases} $$

Then one has

5.12 $$z:=\left(\begin{array}{c} z_{2n}\\ z_{2n+1} \end{array}\right)=\left(\begin{array}{cc} C_{2} & 0\\ 0 & 1 \end{array}\right)\left(\begin{array}{c} v\\ v_{2n+1} \end{array}\right),$$

where

5.13 $$ C_{2}=\operatorname{diag}(\varrho_{1}, \varrho_{2},\ldots,\varrho_{2n}), $$

and

$$ \varrho_{i}=\textstyle\begin{cases} \sqrt{a_{i}}&\text{if } i\in I_{+}\cup I_{-}, \\ 1&\text{if } i\notin I_{+}\cup I_{-}. \end{cases} $$

Combining (5.11) with (5.12) yields

5.14 $$ \bar{g}(v,v_{2n+1})=h(z):=\sum _{i\in I_{+}}z_{i}^{2}-\sum _{i\in\tilde{I}_{-}}z_{i}^{2}. $$

Without loss of generality, suppose that $h(z)>0$. Let

$$ \theta= \biggl( \frac{\sum_{i\in\tilde{I}_{-}}z_{i}^{2}}{h(z)+ \sum_{i\in\tilde{I}_{-}}z_{i}^{2}} \biggr) ^{\frac{1}{2}} = \biggl( \frac{\sum_{i\in\tilde{I}_{-}}z_{i}^{2}}{\sum_{i\in I_{+}}z_{i}^{2}} \biggr) ^{\frac{1}{2}}, $$

then $0\leq\theta<1$. Let

$$ \bar{z}_{i}=\textstyle\begin{cases} \theta z_{i},&i\in I_{+}, \\ z_{i},& \text{otherwise}. \end{cases} $$

This together with the definition of *θ* implies that

5.15 $$\begin{aligned} h(\bar{z}) =&\theta^{2}\sum _{i\in I_{+}}z^{2}_{i}-\sum _{i\in\tilde{I}_{-}}z_{i}^{2} \\ =& \biggl( \frac{\sum_{i\in\tilde{I}_{-}}z_{i}^{2}}{\sum_{i\in I_{+}}z^{2}_{i}} \biggr) \sum_{i\in I_{+}}z^{2} _{i}-\sum_{i\in\tilde{I}_{-}}z_{i}^{2} \\ =&\sum_{i\in\tilde{I}_{-}}z_{i}^{2}-\sum _{i\in\tilde{I}_{-}}z_{i}^{2} \\ =&0. \end{aligned}$$

Using $w=C_{1}v+p_{1}$ and $v=C^{-1}_{2}z_{2n}$, one has $w=C_{1}C ^{-1}_{2}z_{2n}+p_{1}$,

5.16 $$ \bar{w}=C_{1}C^{-1}_{2} \bar{z}_{2n}+p_{1}, $$

and $\bar{v}=C^{-1}_{2}\bar{z}_{2n}$. Applying (5.15), combining (5.10),(5.11) with (5.14) yields

$$ f(\bar{w})=g(\bar{v})=\bar{g}(\bar{v},v_{2n+1})=h(\bar{z})=0. $$

Thus, $\bar{w}\in W^{*}$.

In addition, based on the definition of *z̄* and *θ*, we get

5.17 $$\begin{aligned} \Vert z-\bar{z} \Vert =& \biggl( \sum _{i\in I_{+}}(z_{i}-\bar{z}_{i})^{2} \biggr) ^{\frac{1}{2}} \\ =& \biggl( \sum_{i\in I_{+}}(z_{i}-\theta z_{i})^{2} \biggr) ^{ \frac{1}{2}} \\ =&\frac{(1-\theta^{2})}{1+\theta} \biggl( \sum_{i\in I_{+}}z_{i} ^{2} \biggr) ^{\frac{1}{2}} \\ =&\frac{1}{1+\theta} \biggl( 1-\frac{\sum_{i\in\tilde{I}_{-}}z ^{2}_{i}}{h(z)+\sum_{i\in\tilde{I}_{-}}z^{2}_{i}} \biggr) \biggl( \sum _{i\in I_{+}}z_{i}^{2} \biggr) ^{\frac{1}{2}} \\ =& \frac{h(z)}{(1+\theta) ( \sum_{i\in I_{+}}z_{i}^{2} ) } \biggl( \sum_{i\in I_{+}}z_{i}^{2} \biggr) ^{\frac{1}{2}} \\ =&\frac{h(z)}{(1+\theta) ( \sum_{i\in I_{+}}z_{i}^{2} ) ^{\frac{1}{2}}} \\ =&\frac{h(z)}{ ( \sum_{i\in I_{+}}z_{i}^{2} ) ^{ \frac{1}{2}} +\theta ( \sum_{i\in I_{+}}z_{i}^{2} ) ^{\frac{1}{2}}} \\ =&\frac{h(z)}{ ( \sum_{i\in I_{+}}z_{i}^{2} ) ^{ \frac{1}{2}} + ( \frac{\sum_{i\in\tilde{I}_{-}}z^{2}_{i}}{ \sum_{i\in I_{+}}z^{2}_{i}} ) ^{\frac{1}{2}} ( \sum_{i\in I_{+}}z_{i}^{2} ) ^{\frac{1}{2}}} \\ =&\frac{h(z)}{ ( \sum_{i\in I_{+}}z_{i}^{2} ) ^{ \frac{1}{2}} + ( \sum_{i\in\tilde{I}_{-}}z^{2}_{i} ) ^{\frac{1}{2}}} \\ \leq&\frac{h(z)}{ ( \sum_{i\in I_{+}}z_{i}^{2}+\sum_{i\in\tilde{I}_{-}}z^{2}_{i} ) ^{\frac{1}{2}}} \\ \leq&\frac{h(z)}{h(z)^{\frac{1}{2}}}= \bigl\vert h(z) \bigr\vert ^{\frac{1}{2}}, \end{aligned}$$

where the first inequality comes from the fact that $a^{\frac{1}{2}}+b ^{\frac{1}{2}}\geq(a+b)^{\frac{1}{2}}$, $\forall a,b \in R_{+}$, the second inequality uses assumption $h(z)>0$ and the fact that $h(z)\leq\sum_{i\in I_{+}}z^{2}_{i}+\sum_{i\in\tilde{I}_{-}}z^{2}_{i}$. Using (5.12), (5.16), and (5.17), one has

5.18 $$\begin{aligned} \Vert w-\bar{w} \Vert =& \bigl\Vert \bigl(C_{1}C^{-1}_{2}z_{2n}+p_{1} \bigr)-\bigl(C_{1}C^{-1}_{2} \bar{z}_{2n}+p_{1} \bigr) \bigr\Vert \\ \leq& \bigl\Vert C_{1}C^{-1}_{2} \bigr\Vert \Vert z_{2n}-\bar{z}_{2n} \Vert \\ \leq& \bigl\Vert C_{1}C^{-1}_{2} \bigr\Vert \Vert z-\bar{z} \Vert \\ \leq& \bigl\Vert C_{1}C^{-1}_{2} \bigr\Vert \bigl\vert h(z) \bigr\vert ^{\frac{1}{2}} \\ =& \bigl\Vert C_{1}C^{-1}_{2} \bigr\Vert \bigl\vert f(w) \bigr\vert ^{\frac{1}{2}} \\ =& \sqrt{\lambda_{\max}\bigl(\bigl(C_{1}C_{2}^{-1} \bigr)^{\top}\bigl(C_{1}C_{2}^{-1}\bigr) \bigr)} \bigl\vert f(w) \bigr\vert ^{ \frac{1}{2}} \\ =&\sqrt{\lambda_{\max}\bigl(\bigl(C_{2}^{-1} \bigr)^{\top}\bigl(C_{2}^{-1}\bigr)\bigr)} \bigl\vert f(w) \bigr\vert ^{ \frac{1}{2}} \\ \leq& \bigl\Vert C^{-1}_{2} \bigr\Vert \bigl\vert f(w) \bigr\vert ^{\frac{1}{2}} \\ \leq&\max\biggl\{ 1,\frac{1}{\sqrt{\tilde{\sigma}}}\biggr\} \bigl\vert f(w) \bigr\vert ^{ \frac{1}{2}}, \end{aligned}$$

where the fourth equality comes from the fact that $C_{1}$ is an orthogonal matrix, the last inequality uses the fact that $\|C^{-1} _{2}\|\leq\max\{1,\frac{1}{\sqrt{\tilde{\sigma}}}\}$, and $\tilde{\sigma}>0$ is a constant.

In fact, using the related theory of linear algebra, by the definition of the matrix *Q̂*, one has $\lambda_{i}=\lambda^{M_{1}}_{i}+1$ or $\lambda^{M_{1}}_{i}$ ($i=1,2,\ldots,2n$), where $\lambda^{M_{1}}_{i}$ ($i=1,2, \ldots,2n$) is a real eigenvalue of the matrix $M_{1}$. Set

$$ \tilde{\sigma}=\min \bigl\{ \bigl\vert \lambda^{M_{1}}_{i} \bigr\vert , \bigl\vert \lambda^{M_{1}} _{i}+1 \bigr\vert | \lambda^{M_{1}}_{i}\neq0 \text{ or } \lambda^{M_{1}} _{i}+1\neq0, i=1,2,\ldots,2n \bigr\} >0. $$

Combining (5.13) with (5.9), one has

$$ \varrho_{i}=\textstyle\begin{cases} \sqrt{ \vert \lambda_{i} \vert }&\text{if } i\in I_{+}\cup I_{-}, \\ 1&\text{if } i\notin I_{+}\cup I_{-} \end{cases} $$

and $C^{-1}_{2}=\operatorname{diag}(\varrho^{-1}_{1},\varrho^{-1}_{2},\ldots, \varrho^{-1}_{2n})$, and thus we obtain

$$ \begin{aligned}\bigl\Vert C^{-1}_{2} \bigr\Vert &=\max\bigl\{ \varrho^{-1}_{i} | i=1,2,\ldots,2n\bigr\} \\ &\leq \max\biggl\{ 1, \frac{1}{\sqrt{\tilde{\sigma}}}\biggr\} . \end{aligned}$$

On the other hand, the matrix

$$ M_{1}=\textstyle\begin{cases} (M,-N)^{\top}U^{\top}U(M,-N)+\hat{M}& \text{if } w^{\top}\hat{M}w>0, \\ (M,-N)^{\top}U^{\top}U(M,-N)& \text{if } w^{\top}\hat{M}w=0, \\ (M,-N)^{\top}U^{\top}U(M,-N)-\hat{M}& \text{if } w^{\top}\hat{M}w< 0. \end{cases} $$

Thus, we deduce that *σ̃* is independent of selection of the matrix $M_{1}$. The desired result follows for this case.

*Case* 3. If $J=\emptyset$ and $\tau>0$. Then it follows from

5.19 $$ \bigl\{ w\in R^{2n} | f(w)=0\bigr\} =\bigl\{ w\in R^{2n} | -f(w)=0\bigr\} $$

and (5.10) that

$$ -f(w)=-g(v)=-\sum_{i\in I_{+}}a_{i}v_{i}^{2}+ \sum_{i\in I_{-}}a_{i}v_{i}^{2}+(- \tau). $$

Let $\hat{f}(w)=-f(w)$, $\hat{g}(v)=-g(v)$, $\hat{I}_{+}=I_{-}$, $\hat{I} _{-}=I_{+}$, $\hat{\tau}=-\tau<0$. Then

$$ \hat{f}(w)=\hat{g}(v)=\sum_{i\in\hat{I}_{+}}a_{i}v_{i}^{2}- \sum_{i\in\hat{I}_{-}}a_{i}v_{i}^{2}+ \hat{\tau}. $$

Considering this together with (5.19), using a similar argument to that of Case 2 above, there exist constant $\hat{\sigma}>0$ and $\bar{w}\in W^{*}$ such that

$$ \Vert w-\bar{w} \Vert \leq\hat{\sigma} \bigl\vert \hat{f}(w) \bigr\vert ^{\frac{1}{2}}= \hat{\sigma} \bigl\vert -f(w) \bigr\vert ^{\frac{1}{2}}= \hat{\sigma} \bigl\vert f(w) \bigr\vert ^{\frac{1}{2}}. $$

The desired result follows for this case. □

Now, we give another error bound for the ELCP.

### Theorem 5.2

*Suppose that* $X^{*}$ *is nonempty*. *Then*, *for any* $(x;y)\in R^{2n}$, *there exists a constant* $\rho_{1}>0$ *such that*

$$\begin{aligned} \bigl\Vert (x;y)-(\bar{x};\bar{y}) \bigr\Vert \leq& \rho_{1}\bigl\{ \bigl( \bigl\Vert \min\{ x,y\} \bigr\Vert ^{2}+ \bigl\Vert U(M,-N) (x;y)-Uq \bigr\Vert ^{2}+ \bigl\vert x^{\top}y \bigr\vert \bigr) \\ &{} + \bigl( \bigl\Vert \min\{x,y\} \bigr\Vert + \bigl\Vert U(M,-N) (x;y)-Uq \bigr\Vert + \bigl\vert x^{\top}y \bigr\vert ^{ \frac{1}{2}} \bigr)\bigr\} . \end{aligned}$$

### Proof

By Theorem 5.1, one has

$$\begin{aligned} \Vert w-\bar{w} \Vert \leq&\rho_{0} \bigl( \bigl\vert f(w) \bigr\vert + \bigl\vert f(w) \bigr\vert ^{\frac{1}{2}} \bigr) \\ =& \rho_{0} \bigl\{ \bigl( \bigl\Vert (-w)_{+} \bigr\Vert ^{2}+w^{\top}\bigl[\operatorname{sgn}\bigl(w^{\top} \hat{M}w \bigr)\bigr]\hat{M}w+ \bigl\Vert U(M,-N)w-Uq \bigr\Vert ^{2} \bigr) \\ & {}+ \bigl( \bigl\Vert (-w)_{+} \bigr\Vert ^{2}+w^{\top}\bigl[\operatorname{sgn}\bigl(w^{\top}\hat{M}w\bigr) \bigr] \hat{M}w+ \bigl\Vert U(M,-N)w-Uq \bigr\Vert ^{2} \bigr)^{\frac{1}{2}} \bigr\} \\ \leq& \rho_{0} \bigl\{ \bigl( \bigl\Vert (-w)_{+} \bigr\Vert ^{2}+ \bigl\vert w^{\top}\bigl[\operatorname{sgn}\bigl(w^{ \top}\hat{M}w\bigr)\bigr]\hat{M}w \bigr\vert + \bigl\Vert U(M,-N)w-Uq \bigr\Vert ^{2} \bigr) \\ &{} + \bigl( \bigl\Vert (-w)_{+} \bigr\Vert + \bigl\vert w^{\top}\bigl[\operatorname{sgn}\bigl(w^{\top}\hat{M}w\bigr)\bigr] \hat{M}w \bigr\vert ^{\frac{1}{2}}+ \bigl\Vert U(M,-N)w-Uq \bigr\Vert \bigr) \bigr\} \\ \leq& \rho_{0} \bigl\{ \bigl( \bigl\Vert (-Aw)_{+} \bigr\Vert ^{2}+ \bigl\Vert (-Bw)_{+} \bigr\Vert ^{2}+2 \bigl\vert x ^{\top}y \bigr\vert + \bigl\Vert U(M,-N)w-Uq \bigr\Vert ^{2} \bigr) \\ & {}+ \bigl( \bigl\Vert (-Aw)_{+} \bigr\Vert + \bigl\Vert (-Bw)_{+} \bigr\Vert +\sqrt{2} \bigl\vert x^{\top}y \bigr\vert ^{ \frac{1}{2}}+ \bigl\Vert U(M,-N)w-Uq \bigr\Vert \bigr) \bigr\} \\ \leq& \rho_{0} \bigl\{ \bigl(2 \bigl\Vert \min\{Aw,Bw\} \bigr\Vert ^{2}+2 \bigl\vert x^{\top}y \bigr\vert + \bigl\Vert U(M,-N)w-Uq \bigr\Vert ^{2} \bigr) \\ &{} + \bigl(2 \bigl\Vert \min\{Aw,Bw\} \bigr\Vert +\sqrt{2} \bigl\vert x^{\top}y \bigr\vert ^{ \frac{1}{2}}+ \bigl\Vert U(M,-N)w-Uq \bigr\Vert \bigr) \bigr\} , \end{aligned}$$

where the first equality is by (5.3), the second and third inequalities follow from the fact that

$$ \sqrt{a+b+c}\leq\sqrt{a}+\sqrt{b}+\sqrt{c}\quad \text{for any } a,b,c\in R_{+}, $$

and the fourth inequality is obtained by the fact $(-a)_{+}\leq| \min\{a,b\}|$ for any $a, b \in R$. By the definitions of *A*, *B*, *w*, and setting $\rho_{1}=2\rho_{0}$, we obtain the desired result. □

### Remark 5.1

Obviously, the condition needed in Theorem 5.2 in this paper is strictly weaker than that needed in [4]. The requirements of column monotonicity, $R_{0}$-property, and rank $Q=l$ are removed here. In addition, we establish this global error bound in $R^{2n}$ rather than that in Ω which is defined in [4]. On the other hand, we also present the following examples to compare.

For the ease of description, denote the function used in Theorem 5.2 by

$$\begin{aligned}& \varphi_{1}(x,y)= \bigl\Vert \min\{x,y\} \bigr\Vert ^{2}+ \bigl\Vert U(M,-N) (x;y)-Uq \bigr\Vert ^{2}+ \bigl\Vert x^{ \top}y \bigr\Vert , \\& \varphi_{2}(x,y)= \bigl\Vert \min\{x,y\} \bigr\Vert + \bigl\Vert U(M,-N) (x;y)-Uq \bigr\Vert + \bigl\Vert x^{\top}y \bigr\Vert ^{\frac{1}{2}}, \end{aligned}$$

and denote the function used in Theorem 6 of [4] by

5.20 $$ s(x,y)= \bigl\Vert (-x)_{+} \bigr\Vert + \bigl\Vert (-y)_{+} \bigr\Vert +\bigl(x^{\top}y \bigr)_{+}. $$

### Example 5.1

Consider the ELCP such that

$$M=\left(\begin{array}{ccc} 0 & 1 & 0\\ 0 & 0 & 0\\ 0 & 0 & 1 \end{array}\right),\;N=\left(\begin{array}{ccc} 1 & 0 & 0\\ 0 & 1 & 0\\ 0 & 0 & 1 \end{array}\right),\;K=\left\{\left(\begin{array}{c} 0\\ 0\\ 0 \end{array}\right)\right\}.$$

Its solution set is

$$\begin{aligned} W^{*} =&\bigl\{ (x;y)\in R^{6} \parallel x\geq0,y \geq0,x^{\top}y=0, Mx=y\bigr\} \\ =&\left \{ (x;y)\in R^{6} \bigg| \textstyle\begin{array}{l} x_{1}=x_{3}=0,x_{2}\geq0 , \\ y_{1}=x_{2},y_{2}=0, y_{3}=x_{3}=0 \end{array}\displaystyle \right \} \\ \cup&\left \{ (x;y)\in R^{6} \bigg| \textstyle\begin{array}{l} x_{2}=x_{3}=0,x_{1}\geq0, \\ y_{1}=x_{2}=0,y_{2}=0, y_{3}=x_{3}=0 \end{array}\displaystyle \right \} . \end{aligned}$$

Furthermore, it has no non-degenerate solution [4].

Take $x^{k}=(-k^{-4};k^{2};k^{-1})$, $y^{k}=(k^{2};0;k^{-1})$ with *k* is a positive integer. Denote the closest point in $W^{*}$ by $(\bar{x} _{k};\bar{y}_{k})$. A direct computation gives that $\|Mx^{k}-y^{k}\|=0$, $(\bar{x}_{k};\bar{y}_{k})=(0;k^{2};0;k^{2};0;0)$ as *k* is sufficiently large, and

5.21 $$\begin{aligned} \bigl\Vert \bigl(x^{k};y^{k} \bigr)-(\bar{x}_{k};\bar{y}_{k}) \bigr\Vert =&\bigl[ \bigl(-k^{-4}\bigr)^{2}+0+\bigl(k^{-1} \bigr)^{2}+0+0+\bigl(k ^{-1}\bigr)^{2} \bigr]^{\frac{1}{2}} \\ =&\bigl(k^{-8}+2k^{-2}\bigr)^{\frac{1}{2}}. \end{aligned}$$

Then

$$ \frac{ \Vert (x^{k};y^{k})-(\bar{x}_{k};\bar{y}_{k}) \Vert }{\varphi_{1}(x^{k};y ^{k})+\varphi_{2}(x^{k};y^{k})} =\frac{(k^{-8}+2k^{-2})^{\frac{1}{2}}}{(k ^{-8}+k^{-2})+(k^{-8}+k^{-2})^{\frac{1}{2}}}\rightarrow\sqrt{2} $$

as $k\rightarrow\infty$. Therefore the function $\varphi_{1}(x^{k};y ^{k})+\varphi_{2}(x^{k};y^{k})$ provides an error bound for the point $(x_{k};y_{k})$.

On the other hand, from (5.20), we see that $s(x^{k},y^{k})=k ^{-4}$ for the point $(x_{k};y_{k})$. Then from (5.21), it follows that

$$ \frac{ \Vert (x^{k};y^{k})-(\bar{x}_{k};\bar{y}_{k}) \Vert }{s(x^{k},y^{k})+s ^{\frac{1}{2}}(x^{k},y^{k})}=\frac{(k^{-8}+2k^{-2})^{\frac{1}{2}}}{k ^{-4}+k^{-2}} =\frac{\sqrt{1+2k^{6}}}{1+k^{2}}\rightarrow+\infty $$

and

$$ \frac{ \Vert (x^{k};y^{k})-(\bar{x}_{k};\bar{y}_{k}) \Vert }{s(x^{k},y^{k})}=\frac{(k ^{-8}+2k^{-2})^{\frac{1}{2}}}{k^{-4}} =\sqrt{1+2k^{6}}\rightarrow+ \infty $$

as $k\rightarrow\infty$. Thus, the function $s(x,y)+s^{\frac{1}{2}}(x,y)$ and $s(x,y)$ cannot provide an error bound for the point $(x_{k};y_{k})$.

## Global error bound for special cases of ELCP

In this section, we respectively establish the global error bound of the VLCP and the MLCP based on Theorem 5.2.

### Global error bound for VLCP

Consider the VLCP of finding vector $x\in R^{n}$ such that

$$ Ax+a\geq0, \qquad Bx+b\geq0,\qquad (Ax+a)^{\top}(Bx+b)=0. $$

Denote its solution set by $\hat{X}^{*}$. Certainly, the VLCP is a special case of the ELCP with

6.1 $$M=\left(\begin{array}{c} I\\ 0 \end{array}\right),\;N=\left(\begin{array}{c} 0\\ -I \end{array}\right),\;Q=\left(\begin{array}{c} A\\ B \end{array}\right),\;q=\left(\begin{array}{c} a\\ b \end{array}\right),$$

where $A,B\in R^{m\times n}$, $a, b \in R^{m}$, and $A\neq0$ or $B\neq0$.

Applying Theorem 5.2 to the VLCP, we have the following conclusion.

#### Theorem 6.1

*For the VLCP*, *suppose that* $\hat{X}^{*}$ *is nonempty*. *Then*, *for any* $x\in R^{n}$, *there exists a constant* $\tilde{\rho}_{2} >0$ *such that*

$$\begin{aligned} \operatorname{dist}\bigl(x,\hat{X}^{*}\bigr) \leq&\tilde{ \rho}_{2} \bigl( \bigl\Vert \min\{Ax+a,Bx+b \} \bigr\Vert ^{2}+ \bigl\Vert \min\{Ax+a,Bx+b\} \bigr\Vert \\ &{} + \bigl\Vert (Ax+a)^{\top}(Bx+b) \bigr\Vert + \bigl\Vert (Ax+a)^{\top}(Bx+b) \bigr\Vert ^{\frac{1}{2}}\bigr). \end{aligned}$$

#### Proof

For any $x\in R^{n}$, let $\hat{x}=Ax+a$, $\hat{ y}=Bx+b$. Then

6.2 $$ M\hat{x}-N\hat{y}=Qx+q, $$

where *M*, *N*, *Q* are defined in (6.1). Using the fact that $x=A^{+}b$ is a solution to the linear equation $Ax=b$ if it is consistent, one has

$$ \tilde{U}_{1}(M, -N) (\hat{x};\hat{y})-\tilde{U}_{1}q=0, $$

where $\tilde{U}_{1}=QQ^{+}-I_{2m}={A\choose B}{A\choose B}^{+}-I_{2m}$. Let $\hat{w}=(\hat{x};\hat{y})$. Then the VLCP can be equivalently reformulated as the following ELCP w.r.t. $(\hat{x};\hat{y})$:

6.3 $$ \textstyle\begin{cases} (I,0)(\hat{x};\hat{y})\geq0,\qquad (0,I)(\hat{x};\hat{y})\geq0, \\ ((I,0)(\hat{x};\hat{y}))^{\top}((0,I)(\hat{x};\hat{y}))=0, \\ \tilde{U}_{1}(M, -N)(\hat{x};\hat{y})-\tilde{U}_{1}q=0, \end{cases} $$

and we denote its solution set by $\hat{W}^{*}$. Apply Theorem 5.2 to system (6.3) for $(\hat{x};\hat{y})$. Then there exists $(\hat{x}^{*};\hat{y}^{*}) \in\hat{W}^{*}$ such that

6.4 $$\begin{aligned} \bigl\Vert (\hat{x};\hat{y})-\bigl( \hat{x}^{*};\hat{y}^{*}\bigr) \bigr\Vert \leq& \rho_{2} \bigl\{ \bigl( \bigl\Vert \min\{\hat{x},\hat{y}\} \bigr\Vert ^{2}+ \bigl\Vert \tilde{U}_{1}(M,-N) (\hat{x}; \hat{y})-\tilde{U}_{1}q \bigr\Vert ^{2}+ \bigl\vert \hat{x}^{\top}\hat{y} \bigr\vert \bigr) \\ &{} + \bigl( \bigl\Vert \min\{\hat{x},\hat{y}\} \bigr\Vert + \bigl\Vert \tilde{U}_{1}(M,-N) ( \hat{x};\hat{y})-\tilde{U}_{1}q \bigr\Vert + \bigl\vert \hat{x}^{\top}\hat{y} \bigr\vert ^{ \frac{1}{2}} \bigr) \bigr\} \\ =&\rho_{2}\bigl\{ \bigl\Vert \min\{Ax+a, Bx+b\} \bigr\Vert ^{2}+ \bigl\Vert \min\{Ax+a, Bx+b\} \bigr\Vert \\ &{} + \bigl\Vert (Ax+a)^{\top}(Bx+b) \bigr\Vert + \bigl\Vert (Ax+a)^{\top}(Bx+b) \bigr\Vert ^{\frac{1}{2}}\bigr\} , \end{aligned}$$

where $\rho_{2}>0$ is a constant, the first equality follows from (6.2).

For (6.2), using the fact that $x=A^{+}b$ is a solution to the linear equation $Ax=b$ if it is consistent, one has $x=Q^{+}[(M,-N)( \hat{x};\hat{y})-q]$, $x^{*}=Q^{+}[(M,-N)(\hat{x}^{*};\hat {y}^{*})-q]$, and a straightforward computation gives

$$\begin{aligned} \operatorname{dist}\bigl(x,\hat{X}^{*}\bigr) \leq& \bigl\Vert x-x^{*} \bigr\Vert \\ =&\big\| Q^{+}\bigl[(M,-N) (\hat{x};\hat{y})-q\bigr]-Q^{+} \bigl[(M,-N) \bigl(\hat{x}^{*}; \hat{y}^{*}\bigr)-q\bigr])\big\| \\ \leq& \bigl\Vert Q^{+}(M,-N) \bigr\Vert \bigl\Vert (\hat{x}; \hat{y})-\bigl(\hat{x}^{*};\hat{y}^{*}\bigr) \bigr\Vert . \end{aligned}$$

Combining this with (6.4) and letting $\tilde{\rho}_{2}=\rho _{2}\|Q^{+}(M,-N)\|$ yield the desired result. □

Compared with the error bounds established in [4, 20–23], we remove the assumptions such as monotonicity, positive semidefiniteness, and so on.

### Global error bound for MLCP

Consider the MLCP of finding vector $(x,z)\in R^{n}\times R^{m}$ such that

6.5 $$ x\geq0,\quad Cx+Dz+b\geq0,\qquad x^{\top}(Cx+Dz+b)=0, \qquad Ax+Bz+a=0, $$

where $A\in R^{l\times n}$, $B\in R^{l\times m}$, $C\in R^{n\times n}$, $D \in R^{n\times m}$, $a\in R^{l}$, $b \in R^{n}$. Denote the solution set by $\bar{X}^{*}$. Let $y=Cx+Dz+b$. Then system (6.5) can be rewritten as

6.6 $$ x\geq0, y\geq0, x^{\top}y=0,\qquad Cx-y=-Dz-b,\qquad Ax-0y=-Bz-a. $$

Certainly, the MLCP is a special case of the ELCP with

$$M=\left(\begin{array}{c} A\\ C \end{array}\right),\;N=\left(\begin{array}{c} 0\\ I \end{array}\right),\;Q=\left(\begin{array}{c} -B\\ -D \end{array}\right),\;q=\left(\begin{array}{c} -a\\ -b \end{array}\right).$$

From Theorem 5.2, we have the following conclusion.

#### Theorem 6.2

*For the MLCP*, *suppose that* $\bar{X}^{*}$ *is nonempty*. *Then*, *for any* $x\in R^{n}, z\in R^{m}$, *there exists a constant* $\tilde{\rho}_{3} >0$ *such that*

$$\begin{aligned} \operatorname{dist}\bigl((x;z),\bar{X}^{*}\bigr) \leq& \tilde{\rho}_{3} \bigl\{ \bigl\Vert \min\{x,Cx+Dz+b\} \bigr\Vert ^{2}+ \Vert Ax+Bz+a \Vert ^{2}+ \bigl\vert x^{\top}(Cx+Dz+b) \bigr\vert \\ &{} + \bigl\Vert \min\{x,Cx+Dz+b\} \bigr\Vert + \Vert Ax+Bz+a \Vert + \bigl\vert x^{\top}(Cx+Dz+b) \bigr\vert ^{ \frac{1}{2}} \bigr\} . \end{aligned}$$

#### Proof

By Theorem 5.2, it follows from system (6.6) that, for $(x;y)\in R^{n}\times R^{n}$, there exists a constant $\rho_{3}>0$ such that

6.7 $$\begin{aligned} \bigl\Vert (x;y)-(\bar{x};\bar{y}) \bigr\Vert \leq&\rho_{3} \bigl\{ \bigl( \bigl\Vert \min\{ x,y\} \bigr\Vert ^{2}+ \bigl\Vert U(M,-N) (x;y)-Uq \bigr\Vert ^{2}+ \bigl\vert x^{\top}y \bigr\vert \bigr) \\ &{} + \bigl( \bigl\Vert \min\{x,y\} \bigr\Vert + \bigl\Vert U(M,-N) (x;y)-Uq \bigr\Vert + \bigl\vert x^{\top}y \bigr\vert ^{ \frac{1}{2}} \bigr) \bigr\} , \end{aligned}$$

where $(\bar{x};\bar{y})$ is a solution of system (6.6). For (6.6), using the fact that $x=A^{+}b$ is a solution to the linear equation $Ax=b$ if it is consistent, we know that

$$ Q^{+}\bigl[(M,-N) (x;y)-q\bigr]=z $$

is equivalent to the last two equalities in (6.6). By $U=QQ^{+}-I$, we can get

$$\begin{aligned} U(M,-N) (x;y)-Uq =& Q\bigl[Q^{+}(M,-N) (x;y)-q\bigr]- \bigl[(M,-N) (x;y)-q\bigr] \\ =&Qz-\bigl[(M,-N) (x;y)-q\bigr]. \end{aligned}$$

Combining this with (6.7) and using $y=Cx+Dz+b$, we further obtain

6.8 $$\begin{aligned} \bigl\Vert (x;y)-(\bar{x};\bar{y}) \bigr\Vert \leq&\rho_{3} \bigl\{ \bigl( \bigl\Vert \min\{x,Cx+Dz+b \} \bigr\Vert ^{2} \\ &{} + \bigl\Vert Qz-\bigl[(M,-N) (x;y)-q\bigr] \bigr\Vert ^{2}+ \bigl\vert x^{\top}(Cx+Dz+b) \bigr\vert \bigr) \\ &{} + \bigl( \bigl\Vert \min\{x,Cx+Dz+b\} \bigr\Vert \\ &{} + \bigl\Vert Qz-\bigl[(M,-N) (x;y)-q\bigr] \bigr\Vert + \bigl\vert x^{\top}(Cx+Dz+b) \bigr\vert ^{\frac{1}{2}} \bigr) \bigr\} \\ \leq&\rho_{3} \bigl\{ \bigl( \bigl\Vert \min\{x,Cx+Dz+b\} \bigr\Vert ^{2} \\ &{} + \Vert Ax+Bz+a \Vert ^{2}+ \bigl\vert x^{\top}(Cx+Dz+b) \bigr\vert \bigr) \\ &{} + \bigl( \bigl\Vert \min\{x,Cx+Dz+b\} \bigr\Vert \\ &{} + \Vert Ax+Bz+a \Vert + \bigl\vert x^{\top}(Cx+Dz+b) \bigr\vert ^{\frac{1}{2}} \bigr) \bigr\} . \end{aligned}$$

For any $x\in R^{n}$, $z\in R^{m}$, from $y=Cx+Dz+b$, and using the fact that $x=A^{+}b$ is a solution to the linear equation $Ax=b$ if it is consistent, we obtain that $z=D^{+}(-Cx+y-b), \bar{z}=D^{+}(-C\bar{x}+ \bar{y}-b)$. Combining this with (6.8), one has

6.9 $$\begin{aligned} \bigl\Vert (x;z)-(\bar{x};\bar{z}) \bigr\Vert \leq& \bigl\Vert (x;z)-(\bar{x};\bar{z}) \bigr\Vert _{1} \\ =& \Vert x-\bar{x} \Vert _{1}+ \Vert z-\bar{z} \Vert _{1} \\ =& \Vert x-\bar{x} \Vert _{1}+ \bigl\Vert D^{+}(y-Cx-b)-D^{+}( \bar{y}-C\bar{x}-b) \bigr\Vert _{1} \\ \leq& \Vert x-\bar{x} \Vert _{1}+ \bigl\Vert D^{+} \bigr\Vert \bigl\Vert (y-Cx-b)-(\bar{y}-C\bar{x}-b) \bigr\Vert _{1} \\ \leq& \Vert x-\bar{x} \Vert _{1}+ \bigl\Vert D^{+} \bigr\Vert \bigl(\|(y-\bar{y}\|_{1}+\|C\|\|x-\bar{x} \|_{1}\bigr) \\ =&\bigl(1+ \bigl\Vert D^{+} \bigr\Vert \Vert C \Vert \bigr) \bigl( \Vert x-\bar{x} \Vert _{1}+\|(y-\bar{y}\|_{1}\bigr) \\ \leq&\bigl(1+ \bigl\Vert D^{+} \bigr\Vert \Vert C \Vert \bigr)\sqrt{2n} \bigl\Vert (x;y)-(\bar{x};\bar{y}) \bigr\Vert \\ \leq&\rho_{3}\bigl(1+ \bigl\Vert D^{+} \bigr\Vert \Vert C \Vert \bigr)\sqrt{2n} \bigl\{ \bigl( \bigl\Vert \min\{x,Cx+Dz+b \} \bigr\Vert ^{2} \\ &{} + \Vert Ax+Bz+a \Vert ^{2}+ \bigl\vert x^{\top}(Cx+Dz+b) \bigr\vert \bigr) \\ &{} + \bigl( \bigl\Vert \min\{x,Cx+Dz+b\} \bigr\Vert \\ &{} + \Vert Ax+Bz+a \Vert + \bigl\vert x^{\top}(Cx+Dz+b) \bigr\vert ^{\frac{1}{2}} \bigr) \bigr\} , \end{aligned}$$

where the first and fourth inequalities follow from the fact that $\|x\|\leq\|x\|_{1}\leq\sqrt{n}\|x\|$ for any $x\in R^{n}$. Let $\tilde{\rho}_{3}=\rho_{3}(1+\|D^{+}\|\|C\|)\sqrt{2n}$. Then the desired result follows. □

## Conclusions and remarks

In this paper, we established some new global error bounds for the VLCP and the MLCP based on the global error bound for the ELCP. These global error bounds extend some known results in the literature, which is verified by a numerical comparison.

As the error bound analysis has important applications in the sensitivity analysis and error bound estimation for optimization methods, it would be interesting to investigate whether our new error bound results will give effective global error estimates for some particular methods in solving a non-monotone ELCP (VLCP and MLCP) that does not require any non-degeneracy assumption, such as the Newton-type with quick convergence rate. These will be further considered in the future research.
